# Spraying Ozonated Water on Bobal Grapevines: Effect on Wine Quality

**DOI:** 10.3390/biom10020213

**Published:** 2020-02-01

**Authors:** Ana Campayo, Kortes Serrano de la Hoz, M. Mercedes García-Martínez, M. Rosario Salinas, Gonzalo L. Alonso

**Affiliations:** 1 Universidad de Castilla-La Mancha, E.T.S.I. Agrónomos y de Montes, Cátedra de Química Agrícola, Avda. de España s/n, 02071 Albacete, Spain; ana.campayo@uclm.es (A.C.);; 2 BetterRID (Better Research, Innovation and Development, S.L.), Carretera de Las Peñas (CM-3203), Km 3.2, Campo de Prácticas-UCLM, 02071 Albacete, Spain

**Keywords:** ozonated water, spraying, Bobal, grapevine, wine quality, phenolics, volatiles

## Abstract

Ozonated water is being introduced as an alternative phytosanitary treatment to control grapevine diseases in a context in which the reduction of chemical pesticides has become an urgent necessity. In this study, we evaluated the effect of spraying grapevines with ozonated water on the enological, phenolic, and aromatic qualities of Bobal wines during two consecutive growing seasons. In the first season, ozonated water was applied once during the ripening period on grapevines trained on the traditional gobelet system (S_1_). In the second season, three applications were performed between fruit set and harvest on grapevines grown on a vertical trellis system (S_2_). The S_1_ treatment led to a wine with an increased alcoholic degree and a remarkably higher phenolic content, which resulted in preferable chromatic characteristics. The S_2_ treatment maintained the total phenolic content but significantly enhanced stilbenes and flavanols and also reduced anthocyanins, which negatively affected the wine colour. Regarding aroma, both treatments reduced the content of glycosylated precursors and had different effects on free volatiles, both varietal and fermentative. Thus, the metabolic response of grapevines to the ozonated water stress, and therefore the quality of wines, depended on the ozone dose received by the plants.

## 1. Introduction

Grapevines are among the most important crops worldwide, covering a surface area of 7.4 million hectares [1]. They are affected by several pests and diseases and therefore, to meet qualitative and quantitative production standards, an intensive pesticide schedule is often required [2]. However, environmental and health risks associated with chemical pesticides have encouraged the need to ensure their reasonable use and the search for other approaches for sustainable pest management. In this context, ozonated water is starting to be used as an alternative phytosanitary treatment in the vineyard [3], which has proven to reduce fungal and bacterial populations in grapevines [4,5].

Ozone (O_3_) is a bluish gas with a pungent odour that results from the rearrangement of oxygen atoms when oxygen molecules are subjected to a high-energy input. The oxidation-reduction potential of ozone is 2.07 V, which makes it a powerful oxidizing agent able to attack numerous cellular constituents and inactivate a wide range of bacteria, fungi, viruses, protozoa, and bacterial and fungal spores [6]. The molecule is highly unstable and decomposes spontaneously to oxygen in a very short time. Given its low persistence and spontaneous decomposition to a nontoxic product, ozone is considered environmentally friendly. This gas is partially soluble in water, and its stability in aqueous phase depends on many factors such as the partial pressure of the gas, the temperature, the purity and pH of the water, and the fluid-dynamic conditions [7]. When dissolved in water, ozone is even more unstable than in the gaseous phase. The half-life of gaseous ozone at 20 °C is considered to be 3 days, while in distilled water at the same temperature, it decomposes by 50% within 20 min [8].

Due to its antimicrobial properties and rapid decomposition, ozone has been widely used to extend the shelf life of postharvest fruits and vegetables since its recognition as a safe food disinfectant in 1997 [8]. In postharvest wine grapes, it has also been applied to prevent the use of sulfites during winemaking [9] and to control mycobiota in fresh grapes or during withering [10]. It is well known that abiotic stress modifies the growth and development of all plant organs in grapevines and, at the berry level, induces the accumulation of secondary metabolites, such as polyphenols and volatiles, as a line of defence against cell damage [11]. This elicitor effect has been exploited to increase these types of compounds in table and wine grapes through postharvest treatments with ozone [9,12,13,14,15,16,17,18,19]. Although less widespread, aqueous ozone is starting to be used before harvest to control grapevine diseases, having shown great potential to control the esca-associated fungus *Phaeoacremonium aleophilum* [5] and to reduce bacterial and fungal populations with similar efficacy to chemical treatments [4]. The spraying of ozonated water on Bobal grapevines has recently been demonstrated to modify the composition of grapes, including phenolic and aroma compounds, and therefore their quality [3].

Phenolic compounds are plant secondary metabolites involved in plant resistance. In wines, they are responsible for the colour, astringency, bitterness, body, and aroma. Besides their contribution to the organoleptic properties of wine, they have widely recognized health-promoting properties due to their antioxidant activity [20]. In the presence of biotic and abiotic stresses, phenolic compounds are elicited in both plants and postharvest fruits [21]. Indeed, the use of ozone in postharvest table grapes, in addition to reducing the incidence of decay, has been demonstrated to induce the accumulation of several classes of phenolic compounds [14,15,17,18,22]. The exposure of wine grapes to this gas has also been demonstrated to increase the biosynthesis of phenolic substances [12,19,23], but also to decrease their content [10,23], or even modify the skin cell wall composition and mechanical properties and therefore the extractability of these compounds [9,24,25,26].

The aroma also plays a key role in the quality of grapes and wines. Wine aroma is the result of the contribution of several hundreds of volatile compounds, including those originating in grapes, prefermentative stages, fermentation, and even during ageing or maturation. Those that come from grapes, known as varietal aromas, can occur as free volatile molecules (so-called odour-active compounds) or as aroma precursors (odourless molecules) that during winemaking or ageing can be transformed into odorants. In addition to phenolics, volatile compounds are formed from secondary metabolism in plants and postharvest fruits and are induced under biotic and abiotic stresses as a defence response [27,28]. Although ozone has been considered detrimental for food aroma, which is true for processed food, living plant cells are able to respond to this stressor, and, if it is accurately managed, a positive effect on aroma can occur [28]. In fact, the ozone treatment of grapes prior to winemaking has been demonstrated to change their aroma profile, promoting the synthesis of terpenes and C_6_ compounds [13], favouring glycosylated aroma precursors [13,19], reducing free volatiles in fresh grapes [16,19], maintaining them in withered grapes [16], or increasing fruity flavour in wine [9].

Considering that ozone treatments have induced changes in the composition of grapes when applied both at postharvest and before harvest, especially in terms of phenolic and aroma compounds, our hypothesis is that the quality of wines made from grapevines subjected to ozonated water treatments would be also affected. Thus, the aim of this study was to evaluate the effect of spraying grapevines with ozonated water on the enological, phenolic, and aromatic quality of Bobal wines. This type of treatment is becoming increasingly popular among winegrowers because of the promising results reported on the control of grapevine diseases, but as far as we know, this is the first study on the impact they can exert on winemaking and the quality of the final product.

## 2. Materials and Methods 

### 2.1. Grapevines

The experiments were conducted during the 2015 and 2016 growing seasons in two rainfed plots of *Vitis vinifera* L. cv. Bobal grapevines located in Castilla-La Mancha (Spain), as described by Campayo et al. [3]. The plot used in 2015 was situated in Castillejo de Iniesta (Cuenca) and contained 23-year-old grapevines trained on the traditional gobelet system. The plot used in 2016 was located in Casas de Haro (Cuenca) and contained 17-year-old grapevines grown on a vertical trellis system. A preliminary visual evaluation of the health status of the plots was carried out before the selection of grapevines, especially with regard to trunk diseases. The visual evaluation of the health status was continued during the ozonated water treatments and even after harvest. In addition, weekly control of other diseases and pests such as the grapevine moth using pheromone traps, downy mildew according to the Goidanich model, powdery mildew, botrytis, the leafhopper *Empoasca vitis*, and the spider mite *Tetranychus urticae* Koch was carried out, all of them showing null incidence.

### 2.2. Ozonated Water

A specific prototype designed to be used in agriculture (Nutricontrol, S.L., Cartagena, Spain) was used to generate the ozonated water immediately prior to the grapevine treatments. The prototype was equipped with an electrode to continuously measure in millivolts (mV) the oxidation-reduction potential (ORP) of the water and an ORP indicator and controller to automatically maintain the desired mV. 

### 2.3. Grapevine Treatments

A different ozonated water treatment was applied to the grapevines in each growing season:

- Spraying 1 (S_1_): treatment carried out in 2015 in which ozonated water was applied by spraying leaves only once during the ripening period, which subsequently coincided with 6 weeks before harvest.
- Spraying 2 (S_2_): treatment performed in 2016 in which ozonated water was applied by spraying leaves after the fruit set, at the beginning of veraison, and during the ripening period, which subsequently coincided with 14, 8, and 3 weeks, respectively, before harvest.

The criteria used to define the timing of application were the phenological stages, avoiding critical periods such as flowering, the fruit set, and the week before harvest. In addition, the three applications of the S_2_ treatment were planned to be approximately equidistant and not too close in time. Each treatment was carried out on 15 randomly selected plants according to Campayo et al. [3]. Moreover, 15 untreated plants were used as the control in each season (C_1_ in 2015 and C_2_ in 2016), leaving a buffer row between the treatment and the control to avoid drift effect. The plants under study were visually healthy in order to minimize the effect of pests or diseases on the quality of grapes. They were located in a homogeneous portion of the plots, and the outer rows were avoided. The ORP of the ozonated water throughout the treatments was 800 ± 25 mV, and the volume sprayed per plant and application to cover the entire canopy was approximately 300 mL. The treatments were carried out early in the morning when the environmental temperature was below 20 °C. Grapes were manually harvested when the optimal technological maturity of the control grapes was achieved (most suitable °Baumé/titratable acidity ratio).

### 2.4. Winemaking

In order to evaluate the effect of the ozonated water treatments on wine quality, control (C_1_ and C_2_) and treated (S_1_ and S_2_) grapes were vinified separately and in duplicate. A red winemaking method was followed, but on a different scale each season.

In the first season, approximately 400 g of grapes of each sample (C_1_ and S_1_) was manually destemmed and crushed in duplicate. The resulting grape mass was macerated for 2 h at room temperature and then pressed manually, removing skins and seeds. Then, 200 mL of must was collected for each replicate, which was sulphited with 140 mg/L of potassium metabisulfite and placed in 250 mL round-bottomed flasks with 2 outlets, one for sample extractions and the other to allow the release of fermentation gases. To carry out the alcoholic fermentation, musts were inoculated with 40 g/hL of Lalvin EC1118 active dry yeast, previously rehydrated according to the supplier (Lallemand, Spain), and 40 g/hL of Nutrient Vit (Lallemand, Spain) was added. The flasks were placed over magnetic stirrers to ensure a homogenous fermentation. The orifice through which samples were extracted was covered with a stopper during the fermentation. The alcoholic fermentations were carried out under a controlled temperature (28 °C). The fermentation evolution was followed by daily measurement of °Brix. After 5 days, 5 g/hL of nutrients was added to prevent a stuck fermentation. When the °Brix value was constant at approximately 7, alcoholic fermentation was considered finished. The next day, the lees were removed, and each wine was bottled and frozen at −18 °C until further analysis.

In the second season, approximately 4 kg of grapes of each sample (C_2_ and S_2_) was manually destemmed and crushed in duplicate. Potassium metabisulfite (20 mg/kg) was added to the resulting grape mass, which was placed in 7 L methacrylate tubes for maceration. Grape skins were kept submerged in must with the aid of a plunger. All the tubes were placed in a multitube fermenter (Martínez Solé y Cía, S.A., Villarrobledo, Spain) with a controlled temperature of 24 ± 1 °C. The next day, 25 g/hL of Lalvin EC1118 active dry yeast, previously rehydrated according to the supplier (Lallemand, Spain), was inoculated to carry out the alcoholic fermentation, which took place at 24 ± 1 °C. The next day, 20 g/hL of Nutrient Vit (Lallemand, Spain) was added. During the maceration-fermentation period, the density, °Brix, and temperature were measured, and grape skins were plunged twice per day. When the density and °Brix were constant at approximately 0.995 g/L and 7, respectively, alcoholic fermentation was considered finished. That day, the wines were pressed manually, and the skins and seeds were removed. The malolactic fermentation was induced by adding 1 g/hL of commercial bacteria (Lalvin VP41, Lallemand, Spain) after removing the lees. This was carried out at 18 ± 1 °C in the same multitube fermenter as the alcoholic fermentation. The correct development of the malolactic fermentation was monitored by daily measurement of the pH and the concentrations of malic and lactic acids by HPLC-RID according to Martínez-Gil et al. [29]. Malolactic fermentation was considered finished when the concentration of malic acid was constant. Then, free SO_2_ concentration was corrected to 50 mg/L, and wines were bottled and frozen at −18 °C until further analysis.

### 2.5. Analytical Methods

#### 2.5.1. Wine Enological Parameters

The classical parameters of the wines such as the alcoholic degree (°A), pH, titratable acidity (TA, g/L of tartaric acid), and volatile acidity (VA, g/L of acetic acid) were analysed by Fourier transform-infrared spectroscopy (FT-IR Multispec, TDI, Barcelona, Spain) equipment using the official methods established by the European Union [30] as a reference.

The total phenol index (TPI) of wines was obtained by measuring the absorbance at 280 nm 20 min after diluting the samples with Milli-Q water (1:100) [31]. The chromatic parameters measured were colour intensity, tonality, and CIELAB coordinates. The colour intensity (CI) and tonality (T) were determined by measuring the absorbances at 420, 520, and 620 nm, with CI being the sum of these absorbances (A_420_ + A_520_ + A_620_), and T the ratio of A_420_/A_520_, respectively [32]. CIELAB coordinates were obtained according to the regulations established by the Commission Internationale de l′Eclairage [33], which include L*, a*, b*, C*, and h*. L* represents lightness with values ranging from 0 (black) to 100 (colourless). The terms a* and b* are the colorimetric coordinates, which represent the respective red/green and yellow/blue colour components (a* > 0 red, a* < 0 green, b* > 0 yellow, b* < 0 blue). The parameters C* and h*, calculated as (a*^2^ + b*^2^)^1/2^ and arctan(b*/a*), indicate chroma and hue angle, respectively. The CIELAB parameters were determined by measuring the transmittance from 380 to 780 nm at 5 nm intervals followed by a calculation using the software ′Color of Wines-2001′ (Perkin-Elmer Hispania, Madrid, Spain). Chromatic differences were calculated according to Ayala et al. [34] using the expression ΔE*_ab_ = (Δa*^2^ + Δb*^2^ + ΔL*^2^)^1/2^. Spectrophotometric determinations were made by Lambda 25 UV-Vis equipment (Perkin Elmer, Norwalk, CT, USA) with 1 and 0.1 cm path length cells for TPI and chromatic parameters respectively, but the absorbances were referenced to 1 cm to calculate the latter parameters. Wines were filtered through a PVDF Durapore filter of 0.45 μm (Millipore, Bedford, MA, USA) prior to measuring the chromatic parameters.

The varietal aroma potential index (IPAv) of wines was determined using a commercially available IPAv kit (Teknokroma S.A., Barcelona, Spain). The method is based on that of Salinas et al. [35] but was modified to enable the spectrophotometric determination of the glucose released from the glycosylated aroma precursors by acid hydrolysis. All analyses were conducted in duplicate on each wine replicate (*n* = 2).

#### 2.5.2. Determination of Low-Molecular-Weight Phenolic Compounds by HPLC-DAD

The analysis of the detailed phenolic composition of wines was based on the method described by Pardo-García et al. [36] using an Agilent 1200 high performance liquid chromatograph (HPLC, Palo Alto, CA, USA) equipped with a diode array detector (DAD, Agilent G1315D, Palo Alto, CA, USA) coupled to an Agilent ChemStation (version B.03.01) data-processing station. Separation was performed on a reversed-phase Zorbax-Eclipse XDB-C18 (4.6 mm × 150 mm, 5 μm particle sizes) and a precolumn of the same material at 30 °C. The HPLC grade solvents used were water/formic acid/acetonitrile (97.5:1.5:1 *v*/*v*/*v*) as solvent A and acetonitrile/formic acid/solvent A (78.5:1.5:20 *v*/*v*/*v*) as solvent B. The elution gradient for solvent B was as follows: 0 min, 5%; 2 min, 10%; 7 min, 14.5%; 10 min, 18.5%; 12 min, 20%; 17 min, 20%; 28 min, 30%; 30 min, 30%; 32 min, 50.5%; 38 min, 80%; 40 min, 100%. Before the analysis, wines were filtered through a PVDF Durapore filter of 0.22 μm (Millipore, Bedford, MA, USA) and 20 μL was injected into the chromatographic system. The system was equilibrated with the starting conditions for 10 min prior to injection of the next sample. The flow rate was 0.5 mL/min. 

Identification of phenolic acids, stilbenes, flavanols, flavonols, and anthocyanins was carried out by comparison with their corresponding UV-Vis spectra and retention time of their pure standards (Sigma–Aldrich, Steinheim, Germany). Compounds were identified and quantified at different wavelengths: gallic acid, syringic acid, and flavanols at 280 nm; *t*-caffeic acid and *t*-caftaric acid at 324 nm; vanillic acid at 256 nm; *t*-*p*-coutaric acid and stilbenes at 308 nm; flavonols at 365 nm; and anthocyanins at 520 nm. Quantification was based on calibration curves of the respective standards at five different concentrations (R^2^ > 0.98). Acids *t*-caftaric and *t*-*p*-coutaric were quantified as *t*-caffeic acid and *t*-*p*-coumaric acid, respectively. Flavonols were quantified as quercetin equivalents and the anthocyanins as malvidin 3-*O*-glucoside equivalents. All analyses were performed in duplicate on each wine replicate (*n* = 2).

#### 2.5.3. Determination of Volatile Compounds by SBSE-GC-MS

Wine volatile compounds were determined according to Sánchez-Gómez et al. [37]. Their extraction was carried out by stir-bar sorptive extraction (SBSE), stirring the samples at 500 rpm for 60 min with a polydimethylsiloxane twister bar (PDMS, 10 mm length, 0.5 mm film thickness). Later analysis was performed using an automated thermal desorption unit (TDU, Gerstel, Mülheim and der Ruhr, Germany) mounted on an Agilent 7890A gas chromatograph system (GC) coupled to a quadrupole Agilent 5975C electron ionization mass spectrometric detector (MS, Agilent Technologies, Palo Alto, CA, USA). The GC system was equipped with a fused silica capillary column (BP21 stationary phase, 30 m length, 0.25 mm I.D. and 0.25 μm film thickness) (SGE, Ringwood, Australia), and the carrier gas was helium with a constant column pressure of 20.75 psi.

The stir bars were thermally desorbed in a stream of helium carrier gas at a flow rate of 75 mL/min with the TDU programmed from 40 to 295 °C (held 5 min) at a rate of 60 °C/min in the splitless desorption mode. The analytes were focused in a programmed temperature vaporizing injector (PTV) (CIS-4, Gerstel) containing a packed liner (20 mg tenax TA) held at −40 °C with cryo cooling prior to injection. After desorption and focusing, the CIS-4 was programmed from -40 °C to 260 °C (held for 5 min) at 12 °C/s to transfer the trapped volatiles onto the analytical column. The CIS-4 was operated in the PTV solvent vent mode (purge flow to split vent of 80 mL/min, vent 75 mL/min, and pressure 20.85 psi). The GC oven temperature was programmed to 40 °C (held for 2 min), raised to 80 °C (5 °C/min, held for 2 min), raised to 130 °C (10 °C/min, held for 5 min), raised to 150 °C (5 °C/min, held for 5 min), and then raised to 230 °C (10 °C/min, held for 5 min). The MS was operated in scan acquisition mode (27–300 *m*/*z*) with an ionization energy of 70 eV. The temperature of the MS transfer line was maintained at 230 °C. 

MS data acquisition was carried out in positive scan mode, but to avoid matrix interferences, the MS quantification was performed in the single ion-monitoring mode using the characteristic *m*/*z* values of the volatiles. The identification of the compounds was performed using the NIST library and was confirmed by comparison with the mass spectra and retention time of their pure standards (Sigma–Aldrich, Steinheim, Germany). The standards employed to identify and quantify volatiles were (the numbers in parentheses indicate the *m*/*z* used for quantification): acetovanillone (151), benzyl alcohol (108), citronellol (69), β-damascenone (121), decanoic acid (60), diethyl succinate (101), ethyl acetate (43), ethyl butyrate (88), ethyl decanoate (43), ethyl dihydrocinnamate (104), ethyl hexanoate (101), ethyl lactate (45), ethyl octanoate (101), ethyl vanillate (151), eugenol (164), farnesol (69), geraniol (69), geranyl acetone (43), guaiacol (109), 1-hexanol (56), hexanoic acid (60), hexyl acetate (43), β-ionone (177), isoamyl acetate (43), linalool (71), linalyl acetate (93), 3-methyl-1-butanol (55), nerol (69), nerolidol (69), octanoic acid (60), phenylacetaldehyde (91), 2-phenylethanol (91), 2-phenylethyl acetate (104), and 4-vinylguaiacol (151). 3-Methyl-1-pentanol was used as an internal standard. Quantification was based on calibration curves of the respective standards at five different concentrations (R^2^ = 0.95–0.99). 2-Methyl-1-butanol was quantified together with 3-methyl-1-butanol with the calibration curve of the latter. All analyses were conducted in duplicate on each wine replicate (*n* = 2). The specific contribution of each volatile compound to the overall wine aroma was determined by calculating the odour activity value (OAV) as the ratio between the concentration of the compound and its odour threshold [38].

### 2.6. Statistical Analysis

The statistical analysis of the data was performed using the SPSS statistics software package version 23.0 for Windows (SPSS, Chicago, IL, USA). The mean values were compared using the independent samples t-test. The mean differences were considered statistically significant when the *p*-value < 0.05 (95% confidence interval).

## 3. Results and Discussion

The effect of the ozonated water spraying treatments S_1_ and S_2_ on the enological quality of Bobal grapes has recently been studied [3]; the purpose of this work was to evaluate the impact of these treatments on wine quality. Neither treatment influenced the development of the alcoholic or malolactic fermentations (data not shown). 

### 3.1. Effect on Enological Parameters

The enological parameters of the wines, which include classical and chromatic parameters and aromatic potential, are shown in Table 1. No significant differences were found between the wines from the control and treated grapevines in terms of the classical parameters such as pH, TA or VA; however, the S_1_ wine showed an increased °A value compared to the respective control, which is consistent with the increased sugar content observed in the grapes from which this wine was produced [3]. In a study on grapevine sensitivity to ozone, one of the effects observed after fumigation with high doses was the enhancement of senescence and the loss of photosynthetically active green area of the leaves, with consequent impairment of carbohydrate translocation to the grapes [39]. In addition, the susceptibility of the plants was higher in consecutive years with similar ozone doses [39]. This was not observed in the S_1_ grapes under the assayed conditions, probably because the ozone dose was insufficient, but could be the explanation for the reduction in the sugar content found in the S_2_ grapes [3], although the resulting wine reached an °A value similar to the wine produced with untreated grapes (Table 1).

The ozonated water treatment carried out in the first season also affected the phenolic content: the S_1_ wine had a markedly higher TPI (+131%) than the respective control (Table 1), which is in accordance with the increased value of this index in the initial S_1_ grapes [3]. Phenolic compounds protect plants from biotic and abiotic stressors, and they are elicited when stress factors, ozone in this case, are present [21]. This induction takes place through the activation of several enzymes involved in phenylpropanoid metabolism (phenylalanine ammonia-lyase and 4-coumarate/coenzyme A ligase) following ozone exposure [40]. The S_2_ grapes, however, showed a lower TPI than untreated grapes [3], although such a difference was not detected in the wine (Table 1), possibly because the winemaking conditions, which included maceration with skins and seeds throughout the alcoholic fermentation, favoured the extraction of phenolic compounds that were not released when the grapes were macerated for 4 h to measure the phenolic maturity [3]. In addition, not only does a simple extraction take place during the maceration-fermentation period, but grape phenolics undergo numerous reactions that deeply alter the colour intensity and stability of the wine [41]. The different effects observed on the °A and TPI between the wines of the two vintages could be related to the dose-dependent effect of ozone, which has been described in plants or postharvest fruits exposed to this gas [10,12,18,28,42]. The plants treated in the second season were subjected to more applications, and the exposure of clusters and canopies was also greater because of the training system. In this two-year study, however, the environmental conditions (climate and/or characteristics of the plot) could also have affected the ozone uptake by the plants and, consequently, the attributes of the grapes and wines.

Phenolic compounds are the main substances responsible for the colour of red grapes and wines. The chromatic parameters of treated wines, which are shown in Table 1, were modified in a different way depending on the ozonated water treatment, probably due to the factors mentioned above. In comparison with the respective control, the S_1_ wine showed higher absorbances at the three selected wavelengths, notably at 520 nm, which indicates that there was a greater presence of yellow, red, and blue tonalities. Accordingly, the S_1_ wine presented significantly higher CI and lower T than C_1_, which are associated with better quality and could be related to the higher TPI (Table 1). Similarly, the postharvest ozone fumigation of Petit Verdot grapes, in an attempt to prevent the use of SO_2_, resulted in a wine with a more intense colour that the authors linked to the higher anthocyanin concentration [9]. On the contrary, the S_2_ treatment led to a wine with lower absorbances than the corresponding control at 420 and especially at 520 nm, which means that the ozonated water applied in this manner caused a loss of yellow and red tonalities in the wine. Consequently, the S_2_ wine showed lower CI and higher (but not significant) T than C_2_. Despite the lower CI of the S_2_ wine, it presented a TPI similar to the corresponding control (Table 1), suggesting that the S_2_ treatment had a negative effect on the colour but not on the colourless phenolic compounds. The results concerning the chromatic parameters were positively correlated with the ones obtained for their respective starting grapes [3]. The chromatic characteristics of a wine may be also defined by the CIELAB coordinates L*, a*, b* and their derived magnitudes C* and h*, which are more indicative of the psychological sensation perceived. The effect of the ozonated water treatments on these parameters was evaluated, and the results are shown in Table 1. In comparison with C_1_, the S_1_ wine showed a darker colour, indicated by a lower value of lightness (L*), as well as a higher chroma (C*), which was the consequence of a higher contribution of red (a*) and yellow (b*) colours. The red colour component increased substantially more (+168%) than the yellow one (+144%), and therefore the hue (h*) was lower in S_1_ than in the control wine. These preferable chromatic characteristics are in agreement with the higher CI and lower T values found in S_1_ wine (Table 1) and could be related to the higher TPI (Table 1) and contents of anthocyanins and other phenolic compounds (Table 2). The S_2_ wine, on the contrary, showed similar lightness (L*) and redness (a*) to the control but lower yellowness (b*), and therefore lower chroma (C*) and hue (h*). Differences between the traditional chromatic parameters and CIELAB coordinates are common [43] since only three absorbances are taken into account to measure CI and T, while for the latter, the entire visible spectrum of the wine is considered. With the aim of evaluating the chromatic differences caused by the ozonated water treatments, ΔE*_ab_ was calculated between control wines and those from treated grapevines (Table 1). It was observed that ΔE*_ab_ was much higher between C_1_ and S_1_ than between C_2_ and S_2_. Taking into account that the human eye is able to recognize colour differences of ΔE*_ab_ at around 3.0 CIELAB units in red wines [44], the colour improvement that occurred in the S_1_ wine would be visually perceptible, while the changes detected in the S_2_ wine could be unnoticed.

The aromatic potential of wines, measured as the varietal aroma potential index (IPAv), is presented in Table 1. This index is a global measure of glycosylated aroma precursors, which are non-volatile molecules that come from grapes and under certain conditions can be transformed into odorants. These precursors are comprised of an aglycone (mainly alcohols, terpenoids, and phenols) that is linked to one or more sugar moieties. Both S_1_ and S_2_ treatments significantly decreased the IPAv of the wines and therefore these would have less glycosylated aroma precursors that could be released over time. By contrast, the S_1_ treatment increased this index in grapes [3]. During winemaking, it is well known that the acidic conditions and the yeast glycosidases lead to the hydrolysis of glycosylated precursors and consequent liberation of free volatiles [45]; therefore, the IPAv decline found in the S_1_ wine, compared to the corresponding control, was probably due to a greater release of aglycones during winemaking, which would have a positive impact on the wine varietal aroma. Concerning the S_2_ wine, the IPAv reduction observed may be due to the lower content of glycosylated aroma precursors detected in the starting grapes [3]. The only published information on the effect of ozone on bound volatile compounds concerns postharvest gaseous treatments of wine grapes during dehydration [13,16,19,46], but the impact on these compounds in wine has not been investigated. In two of these studies, ozone has been seen to favour glycosylation [13,19], suggesting that this abiotic stress induces the accumulation of free volatiles that the plant glycosylates as a way of protecting itself from these compounds that can be toxic in high concentrations. Even so, no general trend has been observed in other works, the effect depending on the dose, exposure time, degree of dehydration, grape variety, and compound concerned [13,16,46].

### 3.2. Effect on Phenolic Compounds

The detailed phenolic composition of wines is shown in Table 2. The low-molecular-weight phenolic compounds identified have been grouped into phenolic acids, stilbenes, flavanols, flavonols, and anthocyanins. According to the type of winemaking carried out each season, the phenolic compounds identified in the control and treated wines were found in concentrations within normal ranges for Bobal wines [47]. A greater number of phenolic compounds and higher concentrations of all the families, except the anthocyanins, were found in the wines examined in the second season, probably as a consequence of the longer skin maceration time. The low amount of anthocyanins found in the wines in the second season could be due to the typical loss of these compounds during the latter stages of maceration, which has been attributed to the ionic adsorption by the negatively charged yeast cell walls and lees, adsorption onto bitartrate crystals, incorporation into polymeric pigments, formation of pyranoanthocyanins, and direct anthocyanin degradation through the oxidative cleavage of the heterocyclic C ring [48]. In addition, all these factors appear to be accentuated with longer maceration times, as was the case for C_2_ and S_2_ wines (7 days of maceration-fermentation) compared to C_1_ and S_1_ (2 h of maceration). Moreover, the supposition of the formation of polymeric pigments is more relevant if we consider the chromatograms obtained for C_2_ and S_2_ wines at 280 and 520 nm (Figure S1) in which a broad envelope peak around minute 30, attributable to these compounds according to the literature [49,50], was observed.

Concerning the effect of ozonated water on the phenolic composition, the S_1_ treatment led to a wine with an increased total amount of phenolic acids, flavanols, flavonols, and anthocyanins, which resulted in more than double the total concentration of phenolic compounds of the control wine. This increase is in agreement with the higher TPI and the chromatic characteristics of this wine (Table 1), as well as with the effect observed in the source grapes [3]. The wine from the S_2_ treatment, in comparison with C_2_, showed no significant differences in terms of the total amount of phenolic compounds, which is consistent with the similar TPI found between the wines. In particular, the S_2_ wine presented a higher overall content of stilbenes and flavanols but a lower concentration of anthocyanins than the respective control, while no significant differences in the total content of phenolic acids and flavonols were found. As previously suggested, the increased concentration of stilbenes and flavanols, which are colourless, could explain that the S_2_ wine showed a TPI similar to the control even though the colour (Table 1) and the anthocyanin content were negatively affected. As previously documented, different doses or times of exposure lead to different metabolic changes [42]. Shock or intermittent ozone treatments did not change or even enhanced some phenolic fractions in grapes, while longer exposures significantly decreased the total phenolic, anthocyanin or stilbene contents [10,18]. The assumption of the authors is that these antioxidant compounds produced by the plant against oxidative stress would be oxidized and depleted under long exposure to ozone [10,18]. In our case, the same treatment induced a different response even within the same family of phenolic compounds (Table 1), which was probably related to different antioxidant activities or sensitivity to ozone.

Phenolic acids in grapes and wines include both hydroxybenzoic (HBA) and hydroxycinnamic (HCA) acids. Phenolic acids in grapes, mainly represented by HCAs, are localized in skins and pulps in the form of tartaric esters. Concerning the effect of the treatments on the individual phenolic acids (Table 2), the S_1_ wine showed higher amounts of the detected HBAs (i.e., gallic, vanillic, and syringic acids), as well as increased contents of the tartaric esters of HCAs (i.e., *trans*-caftaric and *trans*-*p*-coutaric acids) compared to its control. Similarly, higher contents of vanillic, *trans*-caftaric, and *trans*-*p*-coutaric acids were found in the S_2_ wine in comparison with C_2_, whereas syringic acid was reduced, and the rest of the phenolic acids detected were unaffected by this treatment. HCAs, in particular caftaric and coutaric acids, increased by 50–100% in Sauvignon Blanc grapes treated with ozone during postharvest (1.5 g/h, 16 h) [19]. However, the same ozone flow during 12 h led to a slight decrease of HCAs in Grechetto grapes compared to the content at harvest [23]. In this work, except for the slight decrease of syringic acid when the S_2_ treatment was applied, the content of phenolic acids in wine was unaffected or even favoured by the ozonated water treatments. On the contrary, lower contents of gallic and *trans*-*p*-coutaric acids were detected in the S_2_ starting grapes in comparison with the control ones [3], suggesting that this more intensive treatment, rather than inhibiting their synthesis, decreased the extractability of these compounds. This assumption was only evident when measuring the phenolic acids in grapes (equivalent to the beginning of the maceration period) [3] since similar or higher concentrations of these acids were found in the finished S_2_ wine compared to the corresponding control (Table 2). In other words, under the winemaking conditions that these wines were produced, a possible greater accumulation of these compounds as a consequence of the S_2_ treatment would prevail over their lower diffusion from grapes. This supposed higher synthesis but lower extractability was already demonstrated in the case of anthocyanins from grapes that received the S_2_ treatment [3]. In this regard, Laureano et al. reported that postharvest table and wine grapes exposed to ozone gas (30 µL/L, 24 h) suffered a skin hardening that resulted in slow extraction kinetics of phenolic compounds during maceration [25].

Regarding the stilbenes analysed (Table 2), which could not be quantified in the wines produced in the first season, the content of both *trans*-resveratrol and its glucoside piceid-*trans*-resveratrol was higher in S_2_ wine than in the corresponding control. These non-flavonoid phenolic compounds are phytoalexins, defensive substances produced by plants in response to various biotic and abiotic stressors such as pathogen attack, UV radiation or ozone exposure [51]. In fact, ozone gas has been demonstrated to elicit the biosynthesis of stilbenes in Scots pine seedlings [52], postharvest table grapes [15,17,18,22], and, depending on the dose and exposure time, postharvest wine grapes [12]. The underlying mechanism of stilbene induction with ozone was revealed in transgenic tobacco plants, where the promoter of grapevine stilbene synthase (STS), the enzyme controlling the synthesis of stilbenes, was rapidly induced after treatment with ozone (0.1 µL/L, 12 h) [51].

In terms of flavanols (Table 2), significantly higher concentrations of (+)-catechin were found in the wines from treated grapevines (S_1_ and S_2_) compared to their controls. (−)-Epicatechin was not detected in the wines produced in the second season, but a higher content of this flavanol was also detected in S_1_ compared to C_1_. An increase in the flavanol content was also observed in ozone-treated seedless table grapes after long-term cold storage and retail display [14]. Similarly, in harvested wine grapes, a significant increase in the (+)-catechin concentration was detected after gaseous ozone treatment [23]. Flavanols are known as the best free radical scavengers found in grapes and wines [23], which would justify their increased presence in ozone-treated grapevines and derived wines.

The content of individual flavonols in wines was also affected by the treatments, as shown in Table 2. Flavonols are yellow grape and wine pigments which contribute to the red wine colour, mainly as copigments, and also have a potent antioxidant activity [53]. The S_1_ wine, compared to its control, showed higher amounts of the 3-*O*-glucuronide plus the 3-*O*-glucoside of myricetin and quercetin, as well as the 3-*O*-glucoside or galactoside of laricitrin, the 3-*O*-glucoside of syringetin, and the aglycone quercetin. The greater presence of flavonols in the S_1_ wine could explain the higher contribution of the yellow colour (A_420_ or b*) compared to the corresponding control wine (Table 1). Concerning the S_2_ wine, higher concentrations of quercetin 3-*O*-galactoside and its aglycone were found in comparison with C_2_. Moreover, a low concentration of kaempferol was detected in this wine, while this flavonol could not be quantified in the case of the control wine. The amount of the other flavonols in the S_2_ wine remained similar to the respective control. As with phenolic acids, lower contents of all the flavonol 3-*O*-glycosides in the starting grapes were found in S_2_ compared to C_2_ [3], while these differences were not observed in the finished wines (Table 2). This reinforces the hypothesis that the S_2_ treatment negatively affected the extractability of phenolic compounds such as phenolic acids and flavonols, but their synthesis was not repressed and could even be enhanced. The use of ozone in postharvest treatments has proven to preserve the flavonol content found at harvest in table grapes [14] and even increase it when applied in nitrogen atmosphere [23]. 

As mentioned above, the S_1_ treatment resulted in a wine containing significantly higher amounts of anthocyanins than its control, whereas the opposite effect was observed in the wine from the S_2_ treatment (Table 2). This could be explained by the lower anthocyanin extractability found in the S_2_ starting grapes [3], which in this case was not solved by the winemaking conditions, and/or a greater depletion of these compounds caused by the more intensive ozone treatment. In particular, the concentrations of all the non-acylated anthocyanins detected (i.e., delphinidin, petunidin, peonidin, and malvidin 3-*O*-glucosides) together with the acetylated peonidin and malvidin 3-*O*-glucosides, the *t*-caffeoylated malvidin 3-*O*-glucoside, and the *p*-coumaroylated petunidin and malvidin 3-*O*-glucosides were increased in the S_1_ wine in comparison with its control. A completely different effect was observed in the S_2_ wine, where the amounts of all the non-acylated anthocyanins detected (i.e., delphinidin, peonidin, and malvidin 3-*O*-glucosides) were decreased. Cyanidin 3-*O*-glucoside, which has been seen to be a very minor anthocyanin in Bobal wines [47], was not detected in any of the wines. Anthocyanins are the main compounds responsible for the red colour of a wine, either through their direct contribution or by reacting with other wine compounds, giving rise to anthocyanin-derived pigments. The higher anthocyanin concentration in S_1_ agrees with the higher red component (a* or A_520_) found in this wine (Table 1). The decrease in the anthocyanin content in the S_2_ wine is consistent with the lower A_520_, but no correlation could be found with the CIELAB parameters since a* did not change with respect to the control (Table 1). This could be due to the higher presence of certain phenolic acids, flavonols, and (+)-catechin, which can act as cofactors in the phenomenon known as copigmentation, with the consequent enhancement of the red colour intensity and a bathochromic shift from reddish to bluish hues [54,55]. In fact, it seems that the extent of copigmentation in wine is determined by the quantity of available cofactors [54]. Moreover, a decrease in h* and the yellow component (b*) was detected in the S_2_ wine, which has been previously described in copigmented wines [55]. This phenomenon would result in the decreased anthocyanin content found in the S_2_ wine, and it would be the reason for the similar red component (a*) between C_2_ and S_2_, which unlike A_520_ was calculated from the entire visible spectrum and therefore is more sensitive to subtle changes.

Table 2 also shows the amount of vitisin A and vitisin B in each wine. These anthocyanin-derived pigments belong to a group called pyranoanthocyanins and are formed mainly during alcoholic fermentation by a reaction between malvidin 3-*O*-glucoside and pyruvic acid or acetaldehyde, respectively [56]. The S_1_ treatment favoured the formation of vitisin B, while the S_2_ treatment resulted in a decrease of this compound but did not affect the vitisin A content. The formation of vitisin-like pyranoanthocyanins was directly related to the content of anthocyanins in wines, in particular malvidin 3-*O*-glucoside (Table 2). In comparison to the genuine anthocyanins, these pigments possess a hypsochromically shifted maximum of absorption which results in more orange hues [56]. Therefore, the effect on the content of vitisin B may be one of the causes that explain the higher or lower contribution of the yellow colour (A_420_ or b*) in S_1_ and S_2_ wines, respectively, compared to their corresponding controls (Table 1). No information has been found on the effect of ozone on this type of phenolic compounds, but their greater or lesser presence in wines seems to be directly related to the positive or negative effect of ozone on malvidin 3-*O*-glucoside and presumably acetaldehyde. This volatile compound appears to be produced in large quantities after abiotic stresses but was not induced in *Populus nigra* leaves exposed to realistic ozone concentrations [57].

### 3.3. Effect on Volatile Compounds

As for the effect of ozonated water treatments on wine aroma, in addition to the glycosylated aroma precursors measured through the IPAv parameter (Table 1), free aroma compounds were analysed. The volatile compounds determined, shown in Table 3, were classified into seven groups: acids, alcohols, acetates, ethyl esters, terpenoids, volatile phenols, and others. The odour activity value (OAV) of each compound in each wine is shown in parentheses, paying special attention throughout the discussion to those compounds whose OAV is higher than 1, due to their greater contribution to the wine aroma. The concentrations of the volatile compounds identified in control and treated wines were consistent with other Bobal wines from La Mancha region [58]. Differences between the two vintages may be due to the type of vinification and even the geographical location, which has been seen to change the varietal character of Bobal wines [59]. In comparison with C_1_, the S_1_ treatment led to an increased total content of terpenoids in wines, while the overall amount of the rest of the families remained unaltered. In addition to the higher concentration of free terpenoids found in the source grapes [3], the positive effect on these volatiles whose origin is the grape could also be explained by the aforementioned greater hydrolysis of glycosidic precursors during winemaking. Therefore, the S_1_ treatment with ozonated water could be used to enhance the varietal aroma of Bobal wines, which is very interesting for a neutral variety. On the other hand, the S_2_ treatment had a different effect on free volatiles since the total concentration of acids decreased and the total concentration of alcohols increased with respect to C_2_. 

Acids, whose group includes decanoic, hexanoic, and octanoic acids, were not affected by the S_1_ treatment (Table 3). By contrast, the S_2_ wines showed lower concentrations of hexanoic and octanoic acids than the control, but they still exceeded their odour thresholds. These volatiles are associated with unpleasant cheesy and rancid odours, but they contribute significantly to the complexity of the wine aroma [65]. Although they can be found in grapes, the fatty acids are predominantly formed by yeasts during alcoholic fermentation, so the lower content of hexanoic and octanoic acids in the S_2_ wine was likely due to the decrease of some of their precursors in the S_2_-treated grapes or their reaction with other wine compounds. The first step in the biosynthesis of fatty acids is the formation of acetyl-CoA, whose main source is glucose [66]. As previously commented, the S_2_ grapes had a lower sugar content and yet gave rise to a wine with the same °A value as the control, so possibly the synthesis of fatty acids was disadvantaged.

Regarding alcohols, the S_1_ treatment only increased the content of benzyl alcohol in relation to the control, but its OAV was barely modified (Table 3). Higher contents of this alcohol, associated with floral aroma, have been detected in both free [16] or glycosylated [19] forms in wine grapes exposed to ozone. Thus, the increase observed in the S_1_ wine could be explained by the greater release of glycosylated aroma precursors that occurred during this winemaking. The grapes from which the S_1_ wine was produced had a significantly higher content of 1-hexanol than the control [3], which then did not result in a higher concentration of this C_6_ alcohol in the corresponding wine (Table 3), probably because during the vinification process it reacted with other wine compounds [66]. Apart from the 1-hexanol present in grapes, this alcohol can also come from the transformation of hexanal, *trans*-2-hexenal, *trans*-2-hexen-1-ol, and cis-2-hexen-1-ol during fermentation [67]. Therefore, the notably lower content of *trans*-2-hexenal in the S_1_ starting grapes [3] could have resulted in less formation of this alcohol during winemaking. In the S_2_ wine, however, benzyl alcohol together with 1-hexanol decreased, whereas the sum of amyl alcohols (2-methyl-1-butanol, and 3-methyl-1-butanol) slightly increased compared to the corresponding control. Benzyl alcohol and 1-hexanol originate mainly from grapes and prefermentative steps, and therefore their lower content in the S_2_ wine could be ascribed to the lower IPAv found in the source grapes or their reaction with other compounds during winemaking. On the contrary, amyl alcohols are formed during fermentation, and their synthesis is linked to yeast amino acid metabolism [66]. Thus, the over-production of these higher alcohols in the S_2_ wine may be attributed to a supposed higher content of the precursor amino acids (isoleucine and leucine) in the treated S_2_ grapes and/or their increased degradation during the vinification process. Concerning 2-phenylethanol, which exceeded its odour threshold in the wines of the second season, none of the treatments significantly modified its content.

The majority of wine esters are formed during fermentation and storage through the esterification of carboxylic acids and contribute to the fruity and floral aromas. There are two main classes of esters in wine: the ethyl esters of fatty acids and the acetate esters of higher alcohols. Acetate esters are formed by enzymatic acetylation of alcohols during fermentation [65]. The total content of acetates was not modified by any of the treatments, although both S_1_ and S_2_ wines showed lower amounts of isoamyl acetate than the corresponding controls. Even so, isoamyl acetate, which is associated with banana nuances, had an OAV above the unit in both treated wines. In addition, the S_1_ wine contained lower contents of hexyl acetate and 2-phenylethyl acetate than C_1_. None of the treatments had a significant effect on ethyl acetate, showing concentrations above its odour threshold in all the wines. The S_2_ treatment did not modify the contents of hexyl acetate and 2-phenylethyl acetate either, but slightly increased linalyl acetate compared to the control. Linalyl acetate is the acetate ester of the monoterpene linalool. Although its increase in the treated S_2_ wine was not significant, terpenes have been demonstrated to reduce ozone damage in plants [27] and to be elicited in postharvest grapes treated with ozone [13].

Ethyl esters are formed by the enzymatically catalysed reaction between ethanol and activated medium- and long-chain fatty acids [66]. Among ethyl esters, seven compounds were identified in the S_1_ wines, while the S_2_ wines also contained ethyl lactate since they were subjected to malolactic fermentation (Table 3). Ethyl butyrate, ethyl hexanoate, and ethyl octanoate, which are related to fruity aromas, showed concentrations above their odour thresholds in all the wines analysed. In the wines from the S_1_ treatment, only ethyl vanillate significantly increased its concentration compared to the control, which is consistent with the markedly higher concentration of vanillic acid found in the S_1_ wine (Table 2). Instead, other ethyl esters such as diethyl succinate, ethyl butyrate, ethyl decanoate, ethyl dihydrocinnamate, ethyl hexanoate, and ethyl octanoate did not show notable differences between the S_1_ wine and the respective control. On the other hand, the S_2_ treatment had a greater influence on this group of compounds, decreasing the concentration of diethyl succinate, ethyl butyrate, ethyl hexanoate, and ethyl octanoate in the wine. The decrease in the concentration of the last two ethyl esters coincides with the lower content found in this wine for the acids from which they derive, i.e., hexanoic and octanoic acids (Table 3). Ethyl decanoate, ethyl dihydrocinnamate, ethyl lactate, and ethyl vanillate were not affected by the S_2_ treatment.

The terpenoid group includes terpenes and C_13-_norisoprenoids. They generally originate in grapes, predominantly as glycosylated precursors, and are involved in the floral and fruity aroma of wines. The S_1_ treatment increased the total content of terpenoids mainly due to a greater presence of farnesol and nerolidol, both doubling the concentrations present in the control wine. Although to a lesser extent, the wine from the S_2_ treatment showed higher contents of citronellol and nerolidol than C_2_. This increase in the content of terpenoids as a consequence of the treatments was already observed in the starting grapes [3]. On the contrary, both ozonated water treatments negatively impacted geraniol, reducing its content in wine by approximately 20% compared to the controls. In grapes, the content of this monoterpene was below the detection limit in C_1_ and S_1_ but showed no significant differences between C_2_ and S_2_ [3]. Thus, the decrease of geraniol in both treated wines could be ascribed to its transformation into other monoterpenes such as linalool by an acid-catalysed chemical reaction during fermentation [68], which in turn could explain the higher linalyl acetate content in the S_2_ wine. The only terpenoids whose concentration exceeded the odour threshold were β-damascenone and β-ionone in the wines of the first season, on which the S_1_ treatment had no relevant effect.

Phenolic substances can also contribute to the odour of wines. These may arise from oak storage, grape-derived glycosides transformed by microbiological or chemical processes, and yeast metabolism of phenolic acids [65]. Table 3 shows the concentrations of the volatile phenols identified in the control and treated wines. None of the treatments significantly modified the guaiacol content, which according to the OAV, was the volatile phenol that individually contributed most to the wine aroma. The S_2_ treatment did not affect the eugenol content either, but S_1_ reduced the concentration of 4-vinylguaiacol by around 50% with respect to the control. This volatile with smoky and clovelike aroma is formed during fermentation by decarboxylation of HCAs present in the must. Since the content of these phenolic acids was higher in the S_1_ wine than in the control (Table 2), the notable decrease of 4-vinylguaiacol could be related to the higher content of other phenolic compounds such as catechin (Table 2), which has been seen to inhibit the activity of the yeast enzyme cinnamate decarboxylase [69]. Other volatile compounds such as acetovanillone and phenylacetaldehyde, identified in the wines produced the first and second season, respectively, showed similar concentrations between the wines from control and treated grapevines.

In the literature, postharvest ozone treatments of wine grapes have led to a significant reduction of free volatiles [16,19] or the over-production of terpenes and C_6_ compounds [13]. In plants, abiotic stresses are supposed to enhance the emission of volatile organic compounds. In response to ozone, it is well known that plants produce isoprene, terpenes, and C_6_ compounds, but the opposite effect has also been observed after exposure to this gas [27]. As our results also suggest, it seems that the effect of ozone on volatile compounds is dependent on the dose, exposure time, and type of compound considered. Terpenoids and C_6_ compounds are associated with a defence function and are generated during berry ripening and prefermentation steps. However, other volatiles are formed during fermentation and later stages, such as the majority of acids, alcohols, and esters found in wines. The changes detected in these volatiles were probably due to the increase or decrease of some of their precursors in grapes in response to the ozonated water or their reaction with other compounds present in the wine, since ozone and derived free radicals have a very short half-life [6] that prevent them from directly impacting winemaking.

## 4. Conclusions

Ozonated water used as an alternative phytosanitary treatment to control grapevine diseases induced a defence response in plants, and particularly in grapes, that altered their metabolism and consequently modified the enological, phenolic, and aromatic qualities of the wines. The treatments with ozonated water carried out in this study did not modify the pH, TA or VA of the wines but, unlike the S_2_ treatment, S_1_ increased the °A value, probably due to the increased sugar content in grapes. The S_1_ treatment caused a remarkable increase in the phenolic content of the wine, which resulted in more than double the content of the corresponding control, specifically in terms of phenolic acids, flavanols, flavonols, anthocyanins, and pyranoanthocyanins. The more intensive S_2_ treatment, which maintained the total phenolic content in the wine, significantly enhanced the concentrations of stilbenes and flavanols but reduced anthocyanins and derived pigments due to a lower extractability and/or supposed greater depletion caused by the increased ozone exposure. The contents of vanillic, *trans*-caftaric and *trans*-*p*-coutaric acids, (+)-catechin, and quercetin, which can act as copigments, were favoured in the wines made from treated grapevines. The effect on the phenolic content led to changes in wine colour, particularly that of the S_1_ wine, which presented preferable chromatic characteristics that would be visually perceptible, while the S_2_ treatment negatively affected the colour of the wine but could be unnoticed by the human eye. Regarding wine aroma, both ozonated water treatments reduced the content of glycosylated precursors, which, in the case of the S_1_ wine, was due to their greater release during winemaking that consequently enhanced its varietal aroma. In fact, the total content of free terpenoids was favoured in the S_1_ wine, mainly due to farnesol and nerolidol. The content of some terpenes such as citronellol and nerolidol was also enhanced in the S_2_ wine, whereas both treatments caused a significant loss of geraniol. The application of ozonated water to grapevines not only affected the varietal aroma of wines, but also indirectly modified the volatiles formed during fermentation. In this regard, both treatments reduced the content of isoamyl acetate and, in addition, the S_1_ wine had lower amounts of other acetates, while the S_2_ wine was richer in amyl alcohols but presented lower contents of total acids and certain ethyl esters. Furthermore, our findings confirm that the metabolic response of grapevines to the ozonated water stress, and therefore the quality of wines, depends on the ozone dose received by the plants, which in this study would be determined by the number of applications, the exposure of clusters and canopies, and the uptake based on the environmental conditions. According to our findings, despite the recent boom in ozonated water treatments in the vineyard, generally for phytosanitary purposes, it would be advisable for the winegrowers to be cautious since their effect is not always positive on the quality of grapes and wines. Depending on the objectives pursued, preliminary tests are recommended to establish the optimum treatment conditions, i.e., the dose of ozonated water and timing of application, for each variety and plot, bearing in mind that climatic conditions can also influence the ozone uptake by the plant. Performed under the appropriate conditions, ozonated water treatments constitute a dual-purpose tool to control grapevine diseases and, in turn, improve the quality of grapes and derived wines.

## Figures and Tables

**Table 1:** Enological parameters including classical and chromatic parameters and aromatic potential of Bobal wines.

Treatments	Season 1		Season 2	
	C_1_	S_1_	C_2_	S_2_
**Classical parameters**				
°A (%v)	12.51 ± 0.08 a	13.40 ± 0.09 b	11.58 ± 0.02 a	11.53 ± 0.10 a
pH	3.38 ± 0.02 a	3.44 ± 0.03 a	3.18 ± 0.01 a	3.18 ± 0.01 a
TA (g/L tartaric acid)	6.58 ± 0.34 a	6.96 ± 0.13 a	5.50 ± 0.08 a	5.24 ± 0.15 a
VA (g/L acetic acid)	0.23 ± 0.05 a	0.26 ± 0.05 a	0.10 ± 0.01 a	0.13 ± 0.01 a
TPI	15.65 ± 0.46 a	36.21 ± 3.89 b	29.31 ± 2.98 a	29.31 ± 0.82 a
**Chromatic parameters**				
A_420_	0.0260 ± 0.0011 a	0.0812 ± 0.0009 b	0.1884 ± 0.0074 b	0.1734 ± 0.0076 a
A_520_	0.0366 ± 0.0015 a	0.1336 ± 0.0013 b	0.3779 ± 0.0065 b	0.3340 ± 0.0150 a
A_620_	0.0078 ± 0.0016 a	0.0177 ± 0.0015 b	0.0885 ± 0.0058 a	0.0832 ± 0.0041 a
CI	0.70 ± 0.01 a	2.32 ± 0.04 b	6.55 ± 0.13 b	5.91 ± 0.26 a
T	0.71 ± 0.00 b	0.61 ± 0.00 a	0.50 ± 0.01 a	0.52 ± 0.00 a
L*	69.85 ± 1.34 b	37.41 ± 0.25 a	20.61 ± 0.81 a	21.03 ± 1.00 a
a*	16.10 ± 0.59 a	43.18 ± 0.35 b	53.47 ± 0.82 a	53.12 ± 1.14 a
b*	10.65 ± 0.41 a	25.95 ± 0.06 b	26.49 ± 0.33 b	23.64 ± 1.06 a
C*	19.30 ± 0.72 a	50.38 ± 0.33 b	59.67 ± 0.86 b	58.15 ± 1.25 a
h*	33.49 ± 0.06 b	31.01 ± 0.15 a	26.36 ± 0.19 b	23.99 ± 0.93 a
ΔE*_ab_		44.94		2.90
**Aromatic potential**				
IPAv	10.33 ± 0.33 b	9.42 ± 0.27 a	11.26 ± 0.61 b	10.11 ± 0.29 a

C_1_: control wine (from untreated grapevines); S_1_: wine from grapevines treated by spraying 1; C_2_: control wine (from untreated grapevines); S_2_: wine from grapevines treated by spraying 2. °A: Alcoholic degree (%v); TA: titratable acidity expressed as g/L of tartaric acid; VA: volatile acidity expressed as g/L of acetic acid; TPI: total phenol index; A_420_: absorbance at 420 nm; A_520_: absorbance at 520 nm; A_620_: absorbance at 620 nm; CI: colour intensity; T: tonality; L*: lightness; a*: red/green colour component; b*: yellow/blue colour component; C*: chroma; h*: hue angle; ΔE*_ab_: chromatic differences between control and treated wines; IPAv: varietal aroma potential index. The mean values (*n* = 2) are shown with their standard deviations. For each parameter and season, different letters indicate significant differences according to the independent samples t-test (*p* < 0.05).

**Table 2:** Phenolic compounds determined in Bobal wines.

Treatments	Season 1		Season 2	
	C_1_	S_1_	C_2_	S_2_
**Phenolic acids (mg/L)**				
Gallic acid	2.15 ± 0.05 a	4.47 ± 0.24 b	23.13 ± 0.28 a	22.45 ± 1.00 a
*trans*-caffeic acid	nd	nd	0.28 ± 0.01 a	0.27 ± 0.03 a
Vanillic acid	1.45 ± 0.06 a	2.75 ± 0.26 b	2.79 ± 0.07 a	3.49 ± 0.18 b
Syringic acid	1.12 ± 0.03 a	1.47 ± 0.05 b	2.39 ± 0.11 b	2.12 ± 0.10 a
*trans*-Caftaric acid	0.09 ± 0.00 a	0.55 ± 0.02 b	11.78 ± 0.28 a	13.84 ± 0.34 b
*trans*-*p*-Coutaric acid	0.70 ± 0.01 a	0.74 ± 0.01 b	2.88 ± 0.04 a	2.96 ± 0.05 b
Σ Phenolic acids	5.51 ± 0.13 a	9.98 ± 0.54 b	43.25 ± 0.70 a	45.13 ± 1.56 a
**Stilbenes (mg/L)**				
*trans*-Resveratrol	nq	nq	0.35 ± 0.01 a	0.44 ± 0.04 b
Piceid-*trans*-resveratrol	nq	nq	1.41 ± 0.02 a	1.89 ± 0.02 b
Σ Stilbenes	-	-	1.77 ± 0.02 a	2.34 ± 0.04 b
**Flavanols (mg/L)**				
(+)-Catechin	0.92 ± 0.06 a	2.29 ± 0.14 b	2.81 ± 0.06 a	3.82 ± 0.23 b
(−)-Epicatechin	0.82 ± 0.02 a	1.35 ± 0.10 b	nd	nd
Σ Flavanols	1.74 ± 0.08 a	3.63 ± 0.22 b	2.81 ± 0.06 a	3.82 ± 0.23 b
**Flavonols (mg/L)**				
Myricetin 3-*O*-galactoside	nd	nd	0.36 ± 0.01 a	0.39 ± 0.04 a
Myricetin 3-*O*-glucuronide+glucoside	1.36 ± 0.15 a	4.28 ± 0.29 b	2.57 ± 0.08 a	2.49 ± 0.11 a
Quercetin 3-*O*-galactoside	nd	nd	0.47 ± 0.01 a	0.55 ± 0.04 b
Quercetin 3-*O*-glucuronide+glucoside	0.69 ± 0.07 a	1.95 ± 0.02 b	5.27 ± 0.09 a	5.85 ± 0.59 a
Laricitrin 3-*O*-glucoside/galactoside	0.15 ± 0.02 a	0.52 ± 0.02 b	0.59 ± 0.01 a	0.54 ± 0.08 a
Kaempferol 3-*O*-glucoside	nd	nd	0.46 ± 0.01 a	0.45 ± 0.03 a
Syringetin 3-*O*-glucoside	0.38 ± 0.01 a	1.04 ± 0.03 b	0.83 ± 0.01 a	0.78 ± 0.05 a
Myricetin	nd	nd	0.14 ± 0.00 a	0.14 ± 0.02 a
Quercetin	0.08 ± 0.00 a	0.40 ± 0.02 b	0.44 ± 0.01 a	0.73 ± 0.04 b
Kaempferol	nd	nd	nq	0.07 ± 0.01
Σ Flavonols	2.66 ± 0.22 a	8.19 ± 0.33 b	11.13 ± 0.19 a	11.99 ± 0.72 a
**Anthocyanins (mg/L)**				
Delphinidin 3-*O*-glucoside	0.76 ± 0.07 a	2.46 ± 0.05 b	0.38 ± 0.04	nq
Cyanidin 3-*O*-glucoside	nd	nd	nd	nd
Petunidin 3-*O*-glucoside	1.64 ± 0.16 a	5.76 ± 0.48 b	nd	nd
Peonidin 3-*O*-glucoside	1.76 ± 0.34 a	4.70 ± 0.15 b	0.57 ± 0.04 b	0.38 ± 0.06 a
Malvidin 3-*O*-glucoside	24.14 ± 0.54 a	60.26 ± 9.56 b	3.22 ± 0.30 b	1.75 ± 0.23 a
Peonidin 3-*O*-(6′-acetyl)-glucoside	1.14 ± 0.04 a	2.16 ± 0.35 b	nd	nd
Malvidin 3-*O*-(6′-acetyl)-glucoside	2.62 ± 0.10 a	5.21 ± 0.88 b	nd	nd
Malvidin 3-(6′-*t*-caffeoyl)-glucoside	0.65 ± 0.01 a	0.88 ± 0.10 b	nd	nd
Petunidin 3-(6′-*p*-coumaroyl)-glucoside	nd	0.71 ± 0.06	nd	nd
Malvidin 3-(6′-*p*-coumaroyl)-glucoside	1.52 ± 0.04 a	3.91 ± 0.67 b	nd	nd
Vitisin A Malvidin 3-*O*-glucoside	nd	nd	0.71 ± 0.02 a	0.70 ± 0.02 a
Vitisin B Malvidin 3-*O*-glucoside	0.63 ± 0.02 a	1.98 ± 0.05 b	2.79 ± 0.07 b	1.68 ± 0.13 a
Σ Anthocyanins	34.87 ± 1.24 a	88.03 ± 11.99 b	7.66 ± 0.41 b	4.51 ± 0.43 a
Σ Phenolic compounds (mg/L)	44.78 ± 1.57 a	109.84 ± 13.03 b	66.62 ± 0.56 a	67.79 ± 2.41 a

C_1_: control wine (from untreated grapevines); S_1_: wine from grapevines treated by spraying 1; C_2_: control wine (from untreated grapevines); S_2_: wine from grapevines treated by spraying 2. The mean values (*n* = 2) are shown with their standard deviations. For each compound and season, different letters indicate significant differences according to the independent samples t-test (*p* < 0.05). nd: not detected; nq: not quantifiable.

**Table 3:** Volatile compounds determined in Bobal wines.

	Odour Threshold (μg/L)	Season 1		Season 2	
		C_1_	S_1_	C_2_	S_2_
**Acids (µg/L)**					
Decanoic acid	1000 [60]	917.37 ± 117.18 a (0.92)	629.89 ± 104.28 a (0.63)	228.43 ± 18.20 a (0.23)	222.59 ± 31.57 a (0.22)
Hexanoic acid	420 [60]	1838.54 ± 125.11 a (**4.38**)	1774.81 ± 294.67 a (**4.23**)	4361.65 ± 409.36 b (**10.38**)	2853.99 ± 463.65 a (**6.80**)
Octanoic acid	500 [60]	2333.49 ± 378.19 a (**4.67**)	1772.37 ± 308.77 a (**3.54**)	1485.23 ± 78.56 b (**2.97**)	1219.95 ± 201.24 a (**2.44**)
Σ Acids		5089.40 ± 620.48 a	4177.06 ± 707.72 a	6075.31 ± 482.37 b	4296.52 ± 688.78 a
**Alcohols (µg/L)**					
Benzyl alcohol	200,000 [61]	213.37 ± 49.79 a (0.00)	327.34 ± 48.39 b (0.00)	206.03 ± 8.89 b (0.00)	187.80 ± 8.51 a (0.00)
1-Hexanol	8000 [38]	618.68 ± 105.73 a (0.08)	551.16 ± 17.21 a (0.07)	558.09 ± 44.06 b (0.07)	438.82 ± 17.32 a (0.05)
2 + 3-Methyl-1-butanol	30,000 [38]	14,048.87 ± 1,246.23 a (0.47)	14,251.55 ± 1,621.15 a (0.48)	228,945.84 ± 13,701.97 a (**7.63**)	255,920.62 ± 9,419.51 b (**8.53**)
2-Phenylethanol	10,000 [38]	7015.40 ± 1,014.62 a (0.70)	7032.57 ± 877.96 a (0.70)	41,080.13 ± 2,184.45 a (**4.11**)	43,320.08 ± 3,807.34 a (**4.33**)
Σ Alcohols		21,896.31 ± 2,366.51 a	22,162.62 ± 2,477.21 a	270,790.09 ± 15,650.77 a	299,867.32 ± 10,858.72 b
**Acetates (µg/L)**					
Ethyl acetate	7500 [38]	25,372.05 ± 867.02 a (**3.38**)	26,918.12 ± 860.79 a (**3.59**)	32,093.83 ± 4,866.11 a (**4.28**)	24,808.05 ± 5,430.47 a (**3.31**)
Hexyl acetate	1500 [61]	6.63 ± 0.23 b (0.00)	3.19 ± 0.06 a (0.00)	1.53 ± 0.17 a (0.00)	1.47 ± 0.25 a (0.00)
Isoamyl acetate	30 [38]	2032.55 ± 62.52 b (**67.75**)	1382.58 ± 94.35 a (**46.09**)	564.59 ± 60.81 b (**18.82**)	426.73 ± 28.87 a (**14.22**)
Linalyl acetate	not found	nd	nd	0.14 ± 0.00 a	0.17 ± 0.01 b
2-Phenylethyl acetate	250 [38]	143.19 ± 8.56 b (0.57)	74.59 ± 19.68 a (0.30)	19.82 ± 2.46 a (0.08)	20.58 ± 3.37 a (0.08)
Σ Acetates		27,554.41 ± 938.34 a	28,378.49 ± 764.52 a	32,679.91 ± 4,929.37 a	25,257.01 ± 5,454.39 a
**Ethyl esters (µg/L)**					
Diethyl succinate	200,000 [61]	152.76 ± 15.64 a (0.00)	156.85 ± 6.27 a (0.00)	353.20 ± 37.32 b (0.00)	244.69 ± 24.37 a (0.00)
Ethyl butyrate	20 [38]	138.29 ± 1.97 a (**6.91**)	144.60 ± 12.86 a (**7.23**)	88.68 ± 9.84 b (**4.43**)	59.13 ± 4.33 a (**2.96**)
Ethyl decanoate	200 [60]	71.14 ± 3.48 a (0.36)	78.43 ± 10.95 a (0.39)	68.10 ± 10.29 a (0.34)	71.74 ± 22.77 a (0.36)
Ethyl dihydrocinnamate	1.6 [60]	0.31 ± 0.03 a (0.19)	0.20 ± 0.05 a (0.12)	0.31 ± 0.02 a (0.20)	0.35 ± 0.04 a (0.22)
Ethyl hexanoate	14 [60]	247.99 ± 5.41 a (**17.71**)	244.54 ± 30.88 a (**17.47**)	348.64 ± 35.75 b (**24.90**)	226.16 ± 20.35 a (**16.15**)
Ethyl lactate	154,000 [61]	nd	nd	8449.52 ± 442.07 a (0.05)	10,202.75 ± 3,161.90 a (0.07)
Ethyl octanoate	5 [60]	172.95 ± 2.67 a (**34.59**)	199.90 ± 25.55 a (**39.98**)	454.57 ± 46.11 b (**90.91**)	316.76 ± 69.58 a (**63.35**)
Ethyl vanillate	990 [62]	72.70 ± 13.56 a (0.07)	108.54 ± 12.52 b (0.11)	36.09 ± 3.24 a (0.04)	42.31 ± 6.49 a (0.04)
Σ Ethyl esters		856.13 ± 22.13 a	933.05 ± 79.61 a	9799.10 ± 415.70 a	11,163.88 ± 3,244.40 a
**Terpenoids (µg/L)**					
Citronellol	100 [61]	17.78 ± 3.07 a (0.18)	21.62 ± 2.71 a (0.22)	6.41 ± 0.22 a (0.06)	7.14 ± 0.53 b (0.07)
β-Damascenone	0.05 [38]	0.35 ± 0.06 a (**7.02**)	0.29 ± 0.04 a (**5.77**)	nd	nd
Farnesol	1000 [63]	112.01 ± 14.77 a (0.11)	227.13 ± 26.84 b (0.23)	17.30 ± 3.10 a (0.02)	21.60 ± 3.70 a (0.02)
Geraniol	30 [38]	7.15 ± 0.23 b (0.24)	5.50 ± 1.08 a (0.18)	8.61 ± 0.45 b (0.29)	6.76 ± 0.27 a (0.23)
Geranyl acetone	60 [38]	0.24 ± 0.02 a (0.00)	0.22 ± 0.01 a (0.00)	0.15 ± 0.01 a (0.00)	0.15 ± 0.02 a (0.00)
β-Ionone	0.09 [60]	0.09 ± 0.02 a (0.99)	0.11 ± 0.03 a (**1.19**)	0.02 ± 0.00 a (0.22)	0.02 ± 0.00 a (0.17)
Linalool	25 [60]	1.18 ± 0.09 a (0.05)	1.32 ± 0.13 a (0.05)	2.75 ± 0.12 a (0.11)	3.04 ± 0.26 a (0.12)
Nerol	15 [61]	5.24 ± 1.20 a (0.35)	6.08 ± 1.08 a (0.41)	nd	nd
Nerolidol	15 [61]	1.36 ± 0.12 a (0.09)	2.77 ± 0.25 b (0.18)	3.64 ± 0.45 a (0.24)	5.43 ± 0.74 b (0.36)
Σ Terpenoids		145.39 ± 17.62 a	265.05 ± 24.39 b	38.88 ± 4.16 a	44.14 ± 5.33 a
**Volatile phenols (µg/L)**					
Eugenol	6 [60]	nd	nd	1.19 ± 0.07 a (0.20)	1.02 ± 0.13 a (0.17)
Guaiacol	9.5 [60]	17.21 ± 6.61 a (**1.81**)	26.87 ± 9.06 a (**2.83**)	53.95 ± 8.82 a (**5.68**)	68.48 ± 15.08 a (**7.21**)
4-Vinylguaiacol	40 [38]	13.17 ± 3.05 b (0.33)	6.51 ± 0.59 a (0.16)	nd	nd
Σ Volatile phenols		30.37 ± 9.28 a	33.37 ± 7.88 a	55.15 ± 8.81 a	69.50 ± 15.20 a
**Others (µg/L)**					
Acetovanillone	1000 [62]	74.66 ± 8.09 a (0.07)	75.16 ± 5.65 a (0.08)	nd	nd
Phenylacetaldehyde	1 [64]	nd	nd	0.55 ± 0.05 a (0.55)	0.65 ± 0.06 a (0.65)

C_1_: control wine (from untreated grapevines); S_1_: wine from grapevines treated by spraying 1; C_2_: control wine (from untreated grapevines); S_2_: wine from grapevines treated by spraying 2. The mean values (n=2) are shown with their standard deviations. For each compound and season, different letters indicate significant differences according to the independent samples t-test (*p* < 0.05). nd: not detected. Odour activity values (OAV) are shown in parentheses for each compound and wine. Bold OAV values indicate the compounds with concentrations above their odour threshold. Reference numbers for the odour thresholds (in wine or synthetic wine) are shown in square brackets for each compound.

## Supplementary Materials

The following are available online at https://www.mdpi.com/2218-273X/10/2/213/s1, Figure S1: (**a**) HPLC-DAD overlaid chromatograms of control (C_1_ and C_2_) and treated (S_1_ and S_2_) Bobal wines at 280 nm; (**b**) HPLC-DAD overlaid chromatograms of control (C_1_ and C_2_) and treated (S_1_ and S_2_) Bobal wines at 520 nm.
