# Supplementary figures and images for: Spraying Ozonated Water on Bobal Grapevines: Effect on Wine Quality
Source: Biomolecules. 2020 Feb 1;10(2):213. doi: 10.3390/biom10020213 (PMC7072419; doi:10.3390/biom10020213)

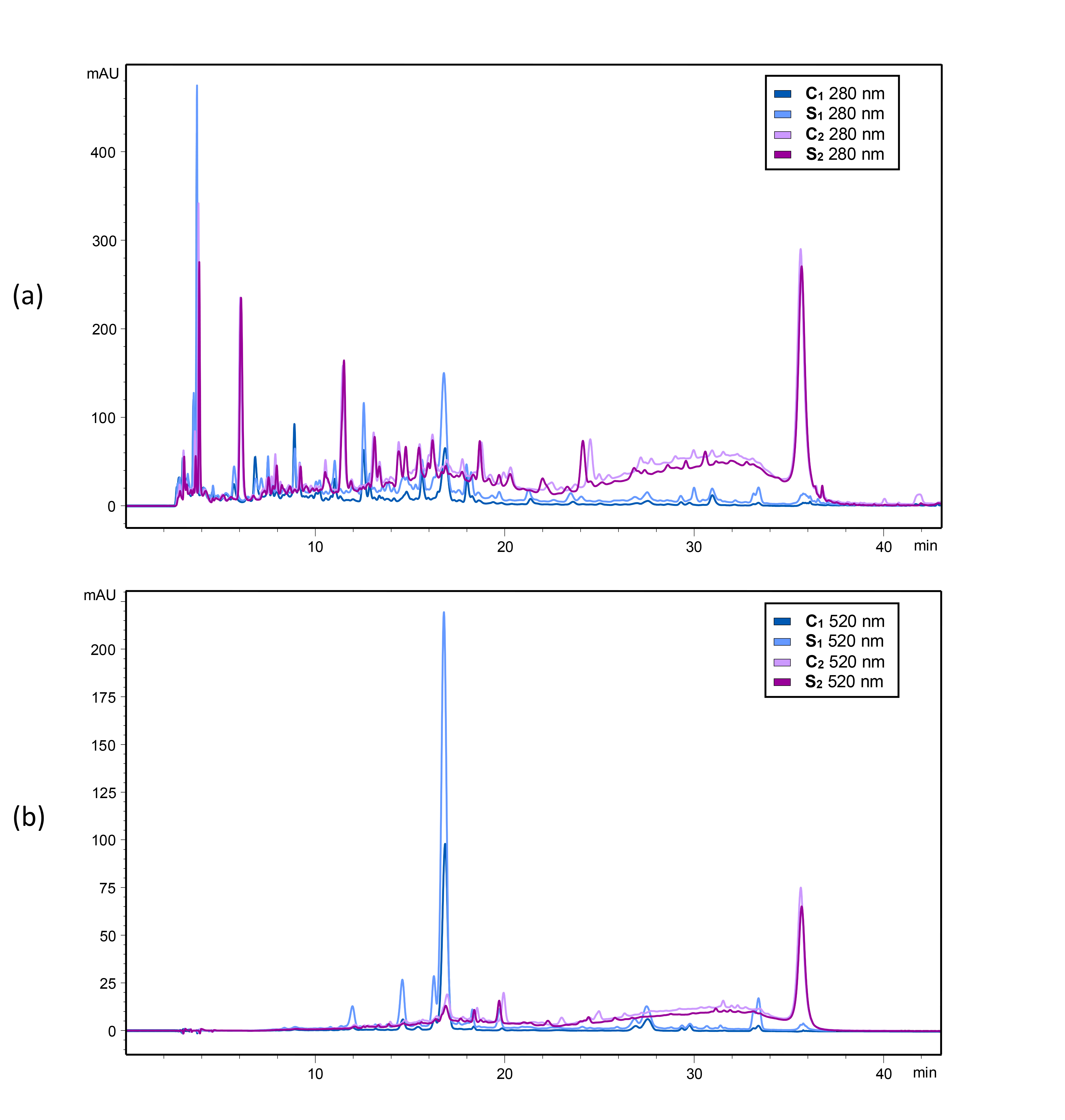

Supplement: Supplementary file 1 [file biomolecules-10-00213-s001.zip › biomolecules-695666-supplementary.jpg]
